# Supplementary figures and images for: BAX activation in mouse retinal ganglion cells occurs in two temporally and mechanistically distinct steps
Source: Mol Neurodegener. 2023 Sep 26;18:67. doi: 10.1186/s13024-023-00659-8 (PMC10521527; doi:10.1186/s13024-023-00659-8)

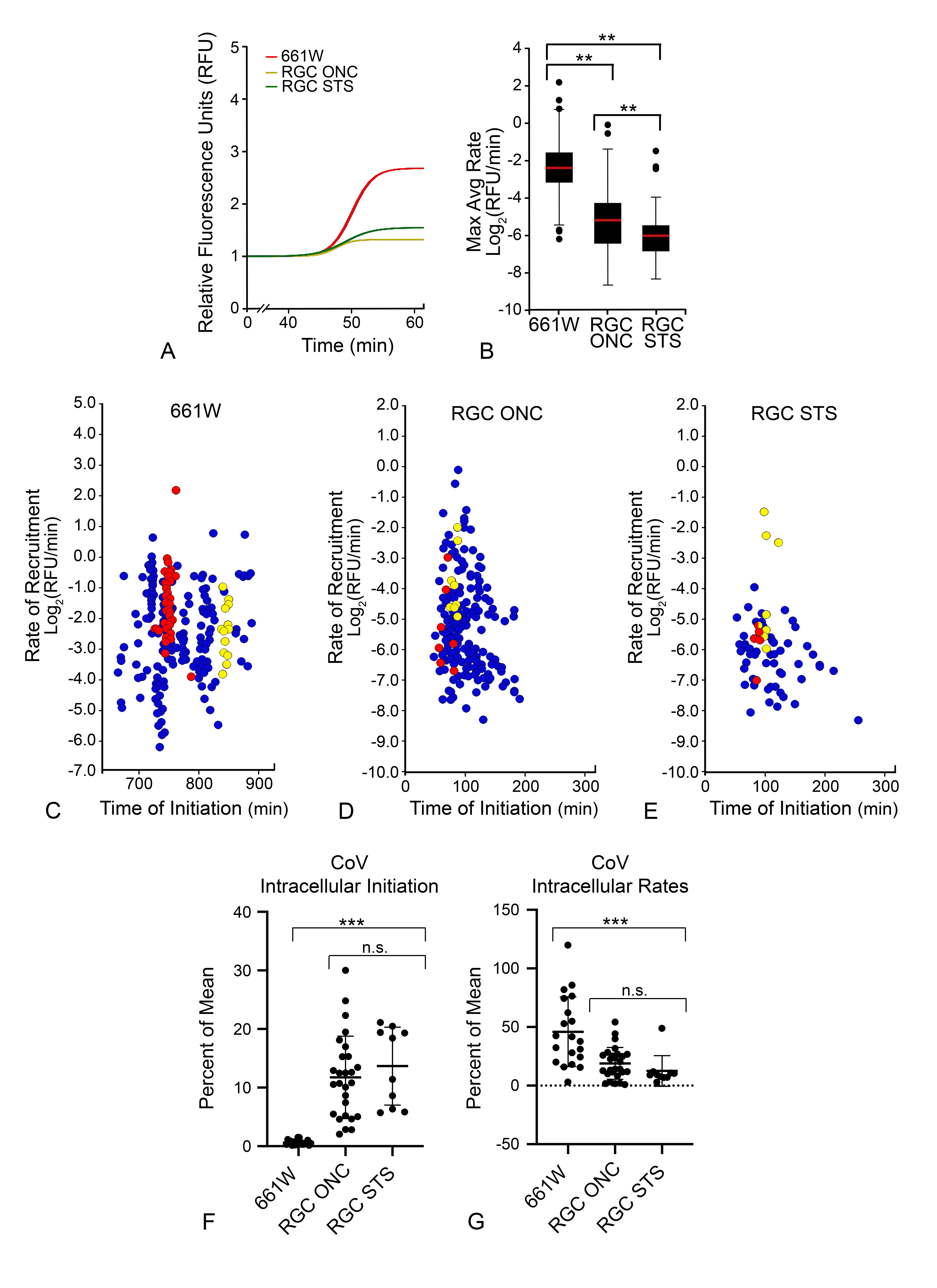

Supplement: Supplementary file 2 — Additional file 2: Supplemental Figure S1. Comparison of BAX translocation kinetics between RGCs and differentiated 661W cells. (A) Graph showing the median curves of GFP-BAX translocation comparing RGCs 7 days after ONC, naïve RGCs exposed to 1 µM staurosporine (STS) during the imaging session and differentiated retinal precursor tissue culture cells (661W) induced for apoptosis by the expression of histone deacetylase 3 (HDAC3) [31]. The time scale (X-axis) has been normalized to show a common time for initiation of BAX accumulation. Raw accumulation curves are shown in supplemental Figure S2. (B) Box and Whisker (5% and 95% limits shown in bars) plots of the maximum rates of GFP-BAX accumulation for all groups shown in (A). The rate of GFP-BAX accumulation in RGC somas is slowest after STS exposure (**P<0.0001, t-test), but both STS and ONC induce a slower rate of accumulation than that observed in 661W cells (**P<0.0001, individual t-tests). Of note, 661W cells exhibit a rate of BAX accumulation that is consistent with multiple different lines of tissue culture cells undergoing apoptosis (Table 1). (C-E) Scatter plots showing the max rate versus time of initiation for individual BAX puncta in the 3 groups. The time scale for graph (C) indicates the time after transfection of differentiated cells with a plasmid expressing human HDAC3. The time scale for graphs (D and E) was set to “0” when animals were euthanized in preparation of explanting the retinas. Puncta from 2 individual cells are plotted as red and yellow circles, respectively. The more stacked the alignment of points relative to the X-axis is indicative of simultaneous initiation of GFP-BAX translocation at all sites within a cell. The broad range of points relative to the Y-axis is indicative of variable rates of translocation at different mitochondrial foci within a single cell. Coefficient of Variation (CoV) analysis of intracellular GFP-BAX kinetics in RGCs exposed to optic nerve crush (ONC) or intr [file 13024_2023_659_MOESM2_ESM.jpg]

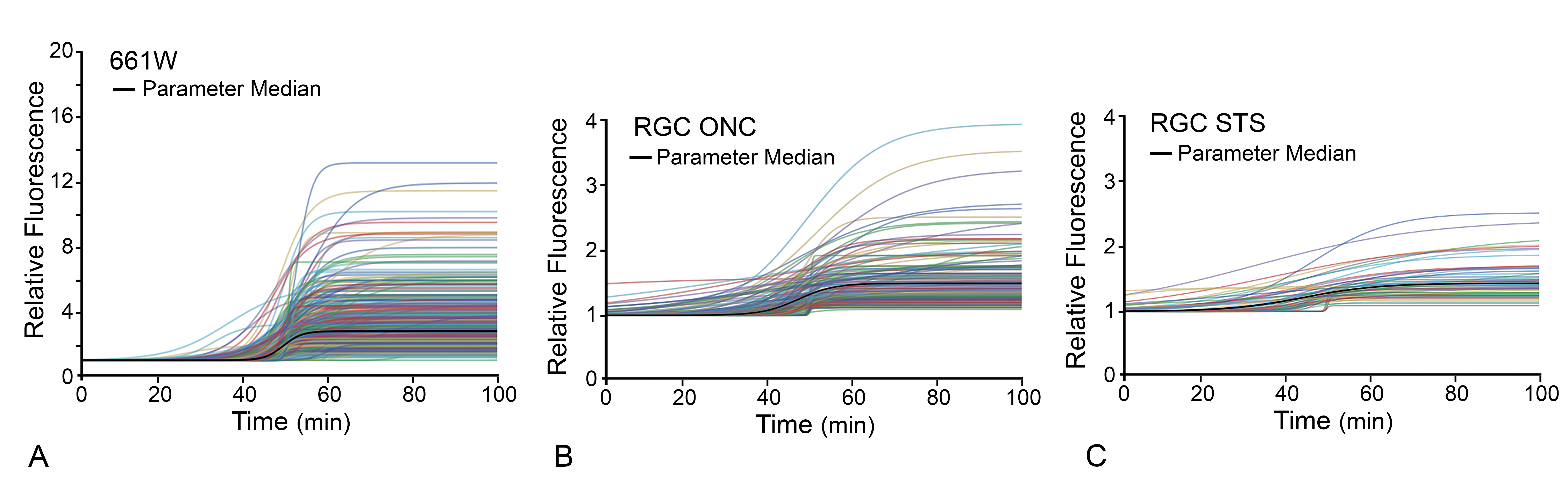

Supplement: Supplementary file 3 — Additional file 3: Supplemental Figure S2. Fitted translocation curves for RGC live-cell imaging. Each curve represents a single mitochondrial locus and is not stratified by individual cell. Median curves of these data are plotted in Supplemental Figure 1A. [file 13024_2023_659_MOESM3_ESM.jpg]

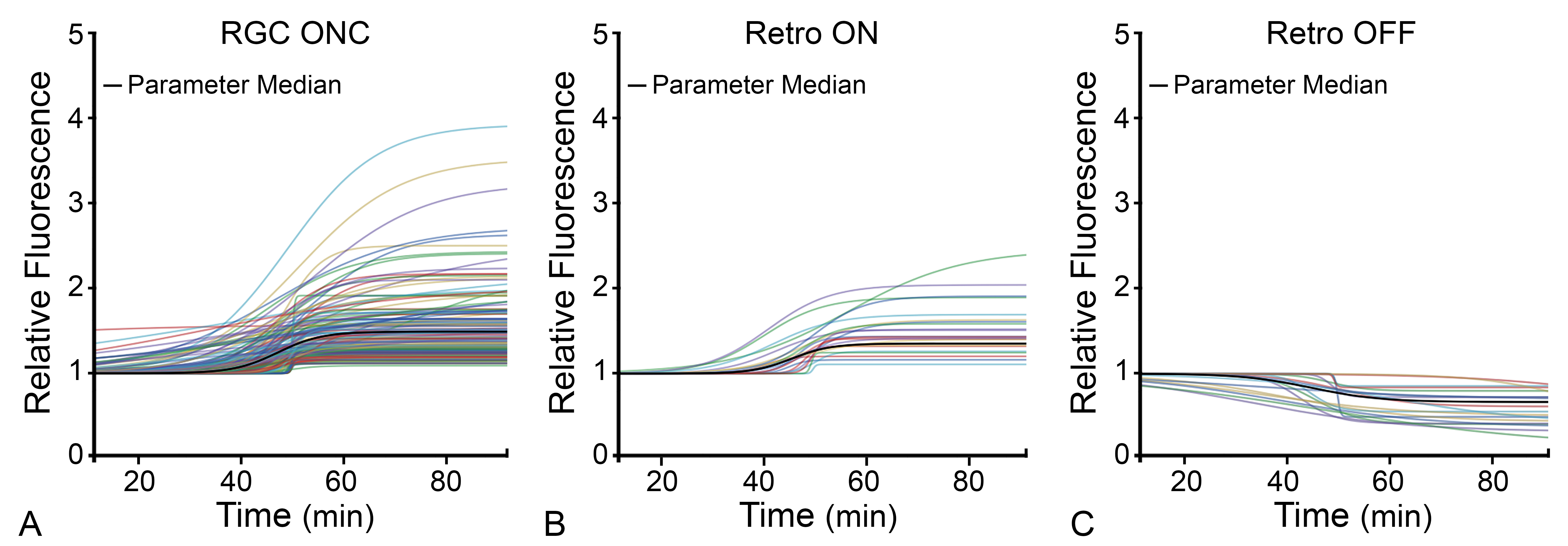

Supplement: Supplementary file 5 — Additional file 5: Supplemental Figure S3. Fitted translocation curves comparing RGCs that exhibit stable translocation of GFP-BAX (A) against the ON rate of translocation in cells that display retrotranslocation (B) and the OFF rate in these cells (C). The data shown in (A) is also graphed in Supplementary Figure S2B. The median curves of these data are plotted in Fig. 4B. [file 13024_2023_659_MOESM5_ESM.jpg]

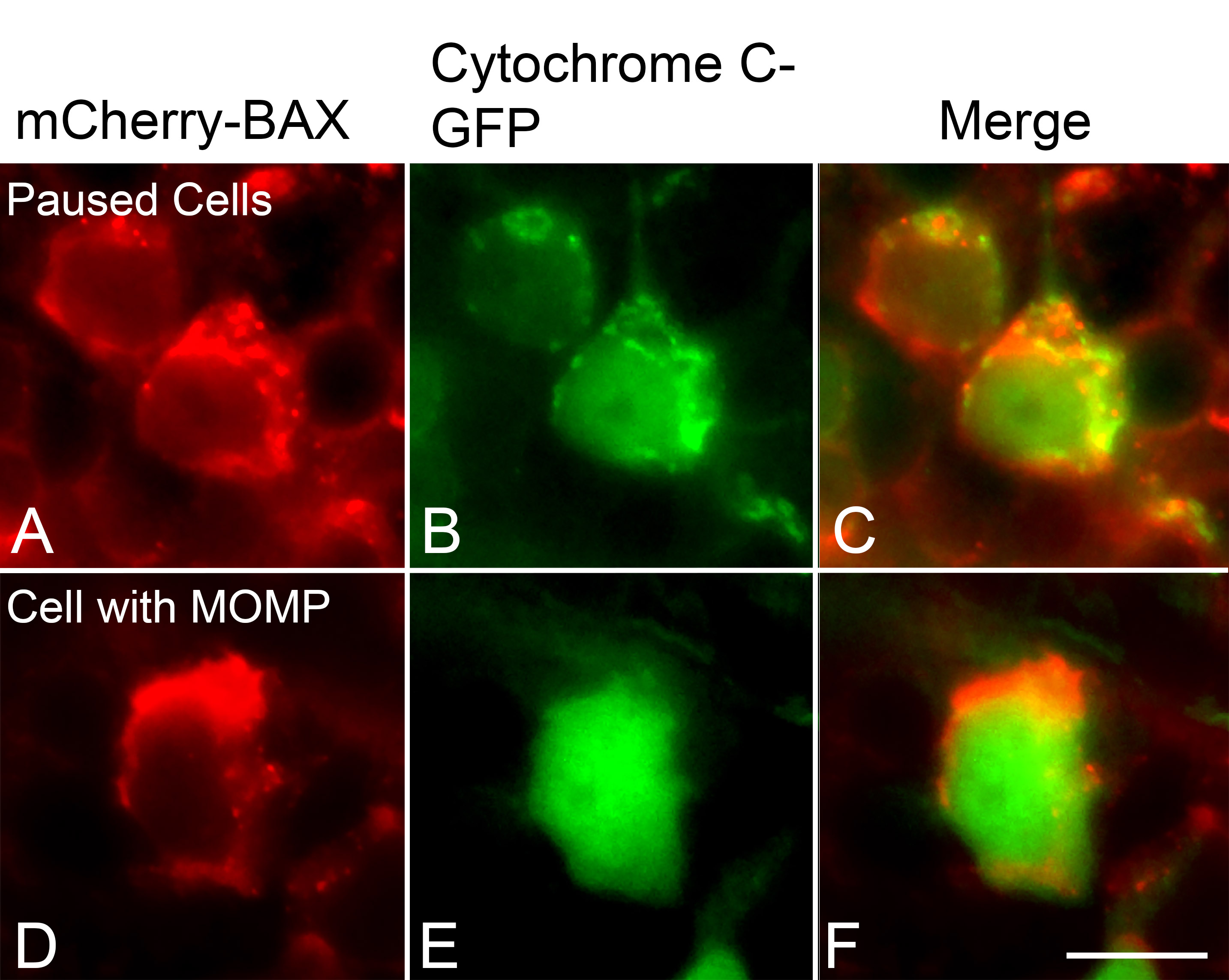

Supplement: Supplementary file 6 — Additional file 6: Supplemental Figure S4. (A-C) Double transduced cell (mCherry-BAX and Cytochrome c-GFP) exhibiting punctate mCherry-BAX and punctate cytochrome c-GFP indicating it has not been released from cellular mitochondria. (D-E) Cell with punctate mCherry-BAX and diffusely localized cytochrome c-GFP indicating mitochondrial outer membrane permeabilization (MOMP). [file 13024_2023_659_MOESM6_ESM.jpg]

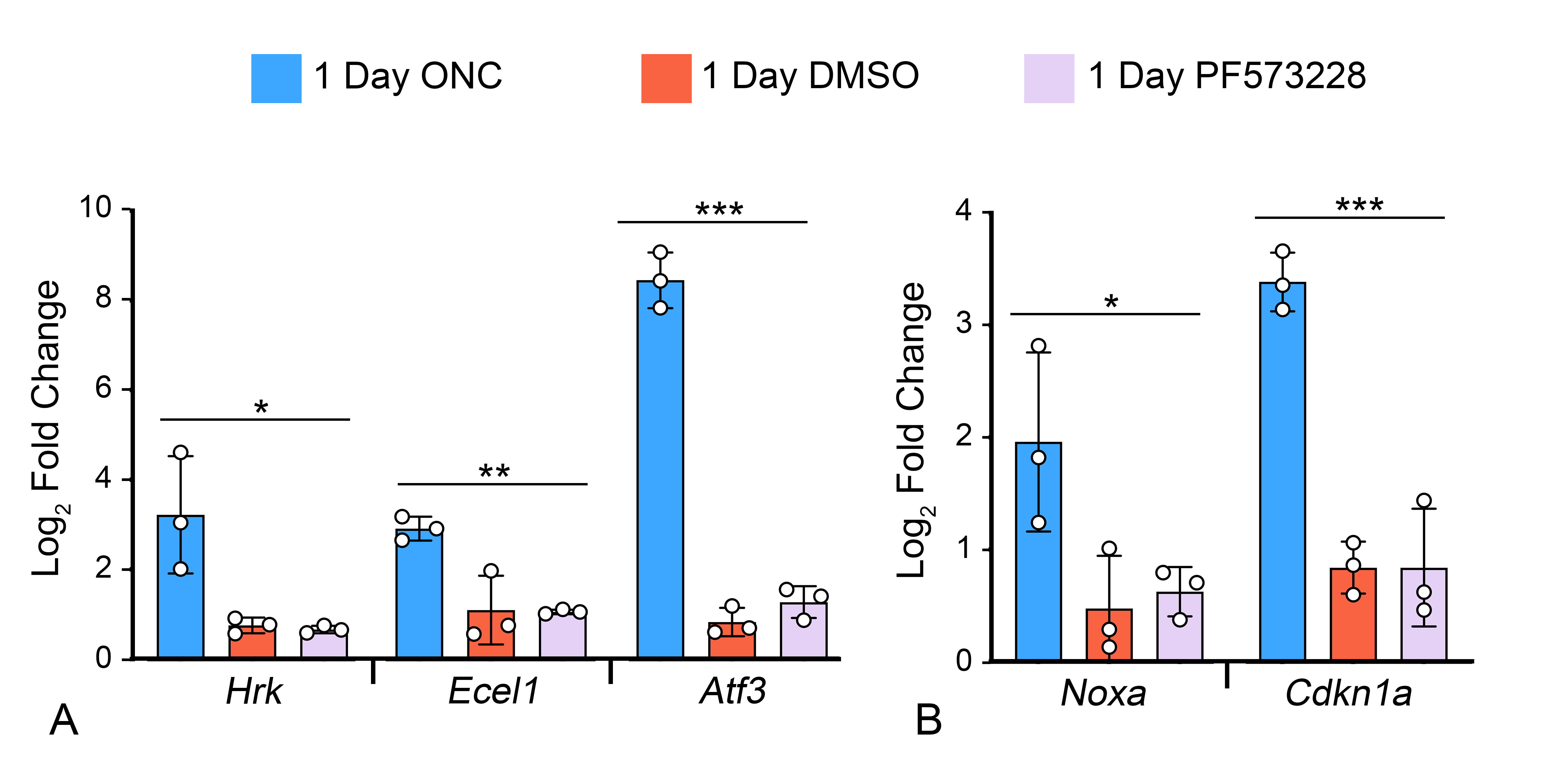

Supplement: Supplementary file 7 — Additional file 7: Supplemental Figure S5. qPCR data for pJUN and p53-associated gene expression comparing 1 day post optic nerve crush and 1 day post intravitreal injection of PF573228. (A) Relative change in transcript abundance for genes regulated by pJUN. (B) Relative change in transcript abundance for genes regulated by p53. The relative change reflects the difference between treated and contralateral eyes for each mouse examined (n=3 experimental replicates of 4 pooled retinas per treatment group). No statistical difference was noted between DMSO and PF573228-injected eyes (t-test comparison of means), while ONC induced significant accumulation of each transcript (ANOVA, * P<0.05, ** P<0.005, *** P<0.0001). [file 13024_2023_659_MOESM7_ESM.jpg]
